# Moyamoya Syndrome in a Child with Neurofibromatosis Type 1: Magnetic Resonance Imaging as a Tool for Clinical Decision Making

**DOI:** 10.7759/cureus.1233

**Published:** 2017-05-09

**Authors:** Jonathan Mayl, Hanisha Patel, Tushar Chandra

**Affiliations:** 1 College of Medicine, University of Central Florida; 2 Radiology, Nemours Chil

**Keywords:** moyamoya, moyamoya disease, moyamoya syndrome, cereberovasculopathy, puff of smoke, ivy sign, mr perfusion

## Abstract

Moyamoya syndrome is a rare cerebrovasculopathy of unknown etiology which is associated with multiple risk factors. Moyamoya was first discovered in Japan and is reported to have an increased prevalence in the Japanese population. The term “Moyamoya” translates into “puff of smoke” and is named after the finding of the collateral cerebral vasculature that develops secondary to occlusion of an internal carotid artery at the entrance into the circle of Willis. This collateral vasculature characterizes the disease. Moyamoya should be included in the differential diagnosis in the pediatric population when a patient presents with stroke or stroke-like symptoms. Diagnosis can be made with catheter angiogram or magnetic resonance angiogram. Recent use of magnetic resonance perfusion imaging has been shown to be useful in clinical decision making while assessing the need for revascularization surgery. We present the case of a 15-year-old with comorbid psychiatric illness, neurofibromatosis type I with brainstem glioma, and Moyamoya syndrome. Considering our patient`s complex medical history of psychiatric illness and previously diagnosed neurofibromatosis, magnetic resonance imaging (MRI) with magnetic resonance angiogram (MRA) and magnetic resonance perfusion proved instrumental in helping rule out the progression of arteriopathy as the cause of his worsening seizures and behavior. In our patient, it was determined that the relative perfusion for each hemisphere of the patient’s brain quantitatively lacked significant differences and he was therefore not a candidate for surgical revascularization. These modalities proved instrumental in surgical decision-making and clinical management of the patient.

## Introduction

Moyamoya syndrome is a chronic progressive cerebrovascular disease that was first described by the Japanese. In fact, “Moyamoya” is a Japanese word meaning 'puff of smoke'. The disease is characterized by unilateral or bilateral stenosis or occlusion of the internal carotid arteries, at the entry point into the circle of Willis, consequently leading to a prominent collateral arterial circulation which often resembles a puff of smoke [1]. Moyamoya is separated into two categories; Moyamoya disease and Moyamoya syndrome. Those with no known risk factors for the characteristic vasculopathy are said to have Moyamoya disease, while those with well-recognized associated conditions to account for the cerebrovasculopathy are classified as having Moyamoya syndrome. The etiology of Moyamoya syndrome is largely unknown, however, some conditions associated with Moyamoya syndrome include neurofibromatosis type 1, down syndrome, and polycystic kidney disease [2]. Some known risk factors for developing Moyamoya include Asian ancestry, radiation therapy to head or neck, down syndrome, and sickle cell anemia. When used alone, the term Moyamoya refers to the distinctive findings on the cerebral angiogram, independent of the cause.

Moyamoya is most prevalent in Japan, but the estimated overall Moyamoya (combined idiopathic Moyamoya disease and Moyamoya syndrome) incidence is in the western United States was found to be of 0.086 per 100,000 with a female to male ratio of two: one [3]. There a is the debate to what the peak age of onset in Moyamoya is, but one study found a bimodal distribution with peaks at five to nine years old and 35 to 39 years old [4]. The clinical manifestations of Moyamoya are variable and include transient ischemic attack (TIA), ischemic stroke, hemorrhagic stroke, and epilepsy. Although there is a debate in this area, a systematic review of population-based studies found that the principal mode of clinical presentation was ischemia, particularly in children [5].

Causes of stroke in childhood include congenital heart disease, sickle cell disease, immune disorders, clotting disorders, head and neck trauma etc. However, Moyamoya should be included in the differential for a stroke in a child. In this disease, repeated ischemic attacks are common. These ischemic events are often secondary to low perfusion areas and hemorrhages originating from the collateral circulation. Multiple areas of cerebral infarction and focal cortical atrophy are frequently found as well [6]. Presentation with an intracranial hemorrhage is more commonly seen in adults. Diagnosis is made with imaging modalities through which intracerebral vasculature can be assessed, such as MR angiogram or catheter angiogram.

The diagnosis of Moyamoya is based upon the characteristic angiographic appearance of stenosis affecting the distal internal carotid artery and proximal circle of Willis' vessels, along with the presence of prominent basal collateral vessels. Definitive diagnosis requires neurovascular imaging such as catheter angiogram, computed tomography (CT) angiogram and magnetic resonance (MR) angiogram [7]. Fluid-attenuated inversion recovery (FLAIR) images demonstrate sulcal hyperintensity and contrast-enhanced images may show leptomeningeal enhancement termed the “ivy sign”. Some studies suggest the ivy sign is correlated with severity of the ischemia and disease progression [8].

Management of Moyamoya syndrome is directed at symptomatic amelioration. Efforts are focused on reducing intracranial pressure, improving cerebral blood flow, and controlling seizures [7]. The American College of Chest Physicians (ACCP) suggests aspirin as initial therapy for children with acute arterial ischemic stroke secondary to Moyamoya. Aside from conservative therapy, surgical revascularization is sometimes an option as well. Generally accepted indications for revascularization include recurrent clinical symptoms due to one) apparent cerebral ischemia or 2) decreased regional cerebral blood flow (CBF), vascular response, and reserve in perfusion studies [7]. There is no cure for Moyamoya and secondary prevention is largely centered on revascularization techniques. The prognosis is variable. However, 66% of patients will have a symptomatic decline over five years [9]. Informed consent statement was obtained for this study.

## Case presentation

A 15-year-old Caucasian male with a medical history of attention deficit hyperactivity disorder (ADHD), neurofibromatosis type 1, and epilepsy presented to our institution. Additionally, this patient also had a known optic nerve glioma as well as a biopsy-proven low-grade glioma of the midbrain, likely secondary to neurofibromatosis type 1. The initial presentation was described as clusters of complex partial seizures, with associated automatisms of lip smacking and left arm raising that usually lasted less than a few minutes followed by a postictal period of increased somnolence. The patient also endorsed rare headaches. It was also noted that the patient was having increasing balance and coordination issues, listlessness, ataxia, and worsening behavior. His speech was slow but fluent and sometimes disjointed. On clinical examination, the upward gaze was slightly restricted and convergence was slow but consistent with his brain stem lesion. He did not have any finger-to-nose ataxia or any major dysmetria. However, he did have very subtle left lower extremity weakness.

**Figure 1:**
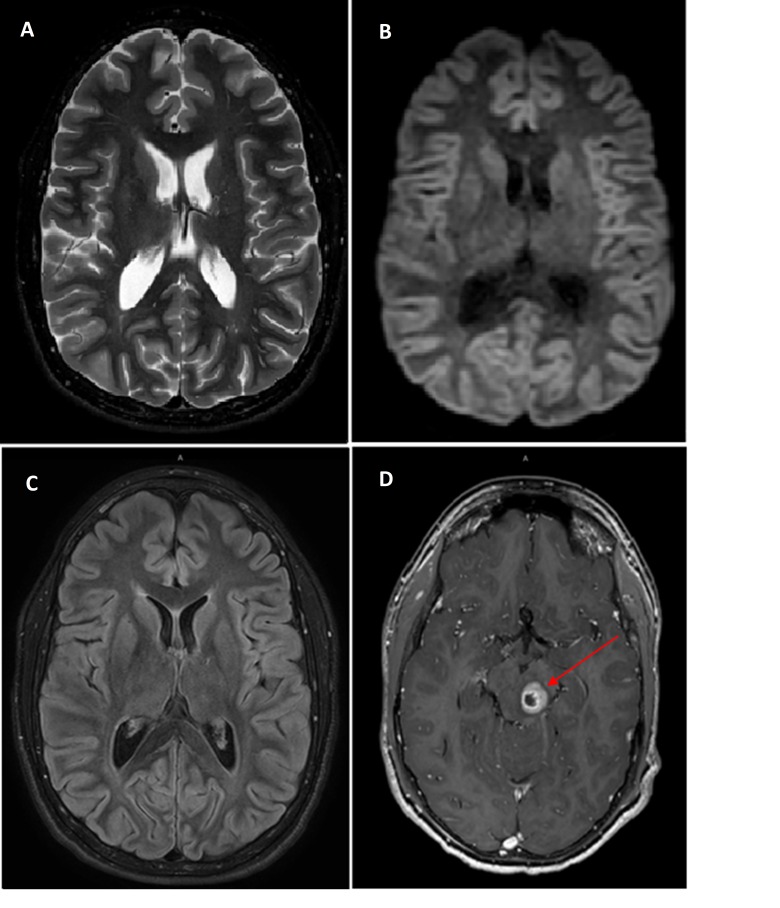
Axial T2 (A), Axial diffusion (B), Axial FLAIR (C) and Post contrast T1 images (D). Post contrast T1 image was obtained after injection of 11.5 ml of gadoterate meglumine (Dotarem) 15-year-old with Moyamoya syndrome and neurofibromatosis type-1 Findings: Magnetic resonance imaging (MRI) sequence demonstrates no acute infraction or ischemia in the brain. Note the absence of “ivy sign” on fluid-attenuated inversion recovery (FLAIR) image. Post contrast T1 image (D) demonstrates a peripherally enhancing mass in left side of midbrain, consistent with a brainstem glioma (red arrow)

**Figure 2:**
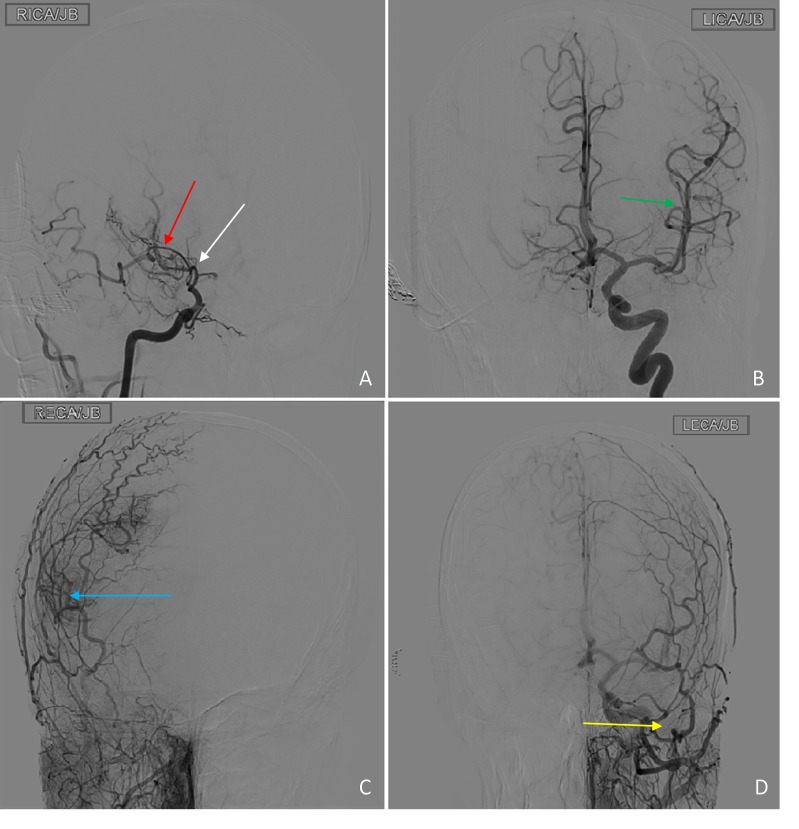
Catheter angiogram in coronal plane after injection of 135 ml of Omnipaque 300 Findings: Catheter angiogram demonstrating severe occlusive changes of the right internal carotid artery (white arrow) with Moyamoya collateralization (red arrow) as well as significant contribution to the anterior circulation via the external carotid artery branches (blue arrow). Normal left internal carotid artery (green arrow) and external carotid artery branches (yellow arrow)

MRI with MR angiogram was obtained in light of these symptoms. MRI demonstrated interval stability of optic nerve glioma (not shown) and a midbrain glioma with no other acute abnormalities (Figure 1A-1D). No sulcal FLAIR hyperintensity or enhancement was noted. Additionally, on MR angiogram, there was stenosis of the right intracranial internal carotid artery with the absence of flow signals of the right middle cerebral artery. Multiple small collaterals were seen in the region of the proximal right middle cerebral artery. The remainder of the anterior and posterior circulation appeared normal. Stenosis of vessels of anterior circulation with collateralization raised suspicion for the possibility of Moyamoya syndrome. After evaluation of these findings, it remained unclear whether these events were related to the patient's psychiatric issues, polypharmacy, high drug levels of phenobarbital, or progression of a possible right-sided Moyamoya syndrome. A catheter angiogram was recommended to further characterize the extent of his Moyamoya vasculopathy. The catheter angiogram with bilateral internal and external carotid artery and selective right vertebral artery injections was performed and this demonstrated severe occlusive changes of the right internal carotid artery with Moyamoya collateralization as well as significant contribution to the anterior circulation via the posterior cerebral artery and contralateral internal carotid artery as well as ipsilateral autosynangiosis (Figure 2A-2C). There were signs of possible mild steal phenomenon of the left cerebral hemisphere secondary to diverted flow through the anterior communicating artery (Figure 2B-2D). Further evaluation with MR perfusion imaging was suggested.

**Figure 3:**
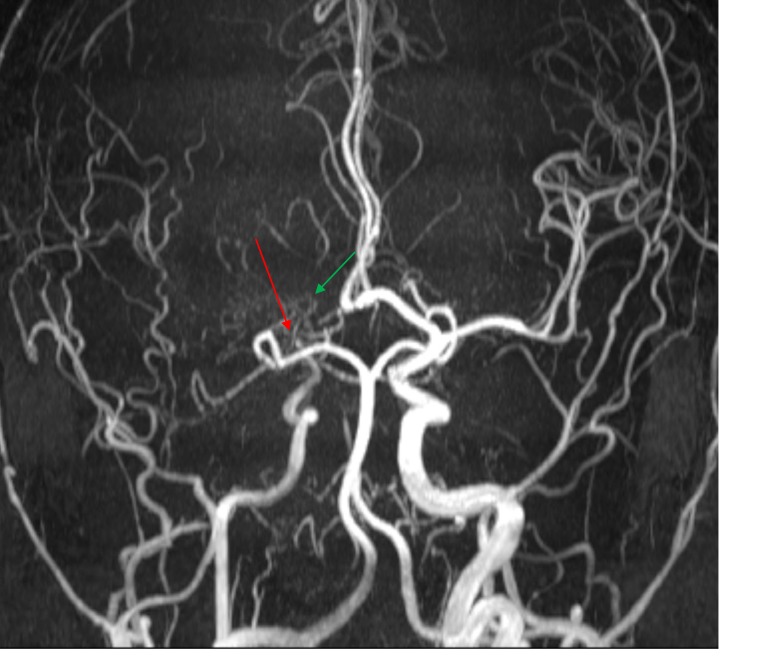
Three dimension (3D) time of flight magnetic resonance (MR) angiogram in coronal plane Findings: Magnetic resonance angiogram demonstrates complete stenosis of the right internal carotid artery at the carotid terminus, just distal to the takeoff of posterior communicating artery (red arrow). The M1 segment of right middle cerebral artery is not visualized secondary to stenosis at the region of middle cerebral artery (MCA). Instead, multiple small collateral vessels are seen in this region (green arrow). M2 and M3 segments of the right middle cerebral artery are formed by collaterals. A1 segment of the right anterior cerebral artery is not seen. Remainder of the right anterior cerebral artery appears normal in caliber and is formed by the anterior communicating artery presumably by flow from the contralateral anterior cerebral artery (ACA) and external carotid artery branches

Subsequently, MRI, MR angiogram, and MR perfusion studies were performed in the same setting. MRI showed no new area of cerebral ischemia or infarction. No intracranial hemorrhage was observed. Brain parenchymal changes were stable from the prior studies. MR angiogram demonstrated complete stenosis of the right internal carotid artery at the carotid terminus, distal to the takeoff of the posterior communicating artery. The M1 segment of the right middle cerebral artery was not visualized secondary to stenosis at the region of middle cerebral artery (MCA). Instead, multiple small collateral vessels were seen in that region. M2 and M3 segments of the right middle cerebral artery were formed by collaterals. An A1 segment of the right anterior cerebral artery was not seen. The remainder of the right anterior cerebral artery appeared normal in caliber and was formed by the anterior communicating artery presumably by flow from the contralateral anterior cerebral artery (ACA). There was a prominent right posterior communicating artery, which supplied the collaterals to the right MCA territory. Multiple prominent collaterals arising from the right external carotid artery were seen supplying the peripheral half of the right cerebral hemisphere (Figure 3). These findings were also unchanged from the prior MR angiogram study.

**Figure 4:**
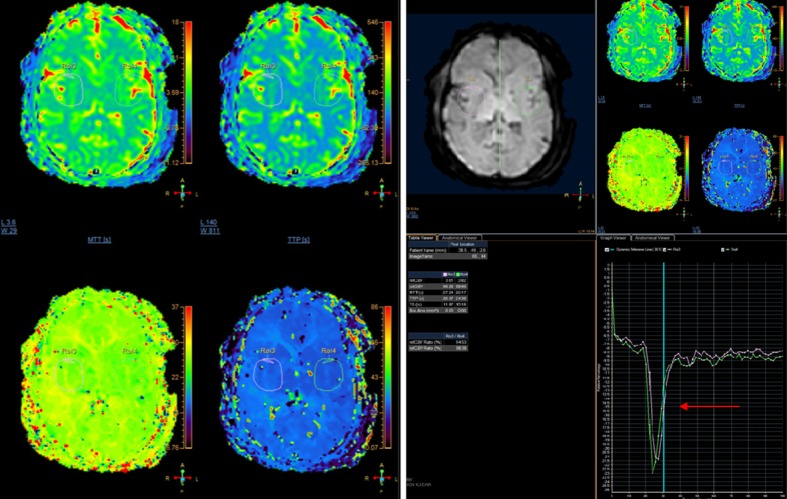
Magnetic resonance perfusion using T2 star technique Findings: No significant difference in relative cerebral blood volume (rCBV) and  relative cerebral blood flow (rCBF) and mean transit time (MTT) values between the two cerebral hemispheres (red arrow). This indicated adequacy of collateral circulation in the right cerebral hemisphere. Technique: Blood flow maps for CBF, CBV and MTT were obtained and values of rCBV, rCBF and MTT were generated after injection of 11.5 ml of gadoterate meglumine (Dotarem). Similar regions of interest (ROIs) were drawn in both cerebral hemispheres in the middle cerebral artery (MCA) territory and corresponding values of rCBV, rCBF and MTT were obtained quantitatively. Additionally, qualitative color maps were obtained for assessment of these parameters

MR perfusion was then performed using a dynamic susceptibility contrast (DSC) T2 star technique after injection of gadolinium-based contrast. Blood flow maps for cerebral blood flow (CBF), cerebral blood volume (CBV) and mean transit time (MTT) were obtained and values of relative cerebral blood volume (rCBV), relative cerebral blood flow (rCBF), and MTT were generated. Similar regions of interest (ROIs) were drawn in both cerebral hemispheres in the MCA territory and corresponding values of rCBV, rCBF, and MTT were obtained quantitatively. Additionally, qualitative color maps were obtained for assessment of these parameters. There were no significant quantitative differences in rCBV, rCBF, and MTT values between the two cerebral hemispheres. Additionally, on the color maps, there were no significant differences between the two hemispheres qualitatively. Furthermore, when perfusion parameters of the cerebellum were considered as the internal reference standard (as the posterior circulation was normal on MR Angiogram), no significant differences were seen in perfusion of either of the cerebral hemispheres with respect to the cerebellum (Figure 4). This indicated adequacy of collateral circulation in the right cerebral hemisphere, the hemisphere that was supplied by the stenosed internal carotid artery (ICA). Based on these findings, the neurosurgery service recommended that revascularization surgery is not required and the patient was kept on medical therapy. A careful lab evaluation demonstrated that the patient's blood level of phenobarbital was high and therefore, it was inferred that the change in the patient’s symptoms was related to excess phenobarbital. The patient’s dose was readjusted. Gradually, the patient’s clinical symptoms improved and approximately two years later, the patient continues to do well with medical management.

## Discussion

Patients presenting with confounding comorbidities are often difficult to manage. In our case, the combination of a psychiatric component as well as an underlying organic condition and polypharmacy made it difficult to clinically determine which symptoms were attributed to the psychiatric component as opposed to which symptoms were attributed to an organic component. This case exemplifies the value of imaging which proved vital in clinical decision making to obviate an invasive surgical procedure in a complex patient. MRI with MR angiogram and MR perfusion play a vital role in assessment for cerebrovascular changes, as illustrated in our case. MRI of the brain provided information about the status of brain parenchyma and ruled out intracranial hemorrhage, infraction or ischemia (Figure 1A, 1B, 1D). The absence of ivy sign on FLAIR images helped exclude formation of leptomeningeal collaterals (Figure 1C). MR angiogram provided a non-invasive visualization of the status of the intracranial vasculature and was especially useful for assessment of the degree of stenosis of the internal carotid artery and the presence of collateral flow (Figure 3). MR perfusion provided a powerful imaging modality to assess the relative quantitative regional parameters, such as cerebral blood flow (CBF), cerebral blood volume (CBV), mean transit time (MTT) and others that contributed clinically useful information in determining the need for revascularization surgery [10]. Because of MR perfusion in combination with FLAIR imaging and the absence of an ivy sign, we could determine that our patient had adequate cerebral vascular perfusion that was relatively equal in both hemispheres of the brain. This information helped us to exclude an etiology of cerebral vasculopathy and consequently lead to the evaluation of other avenues including comorbidities and pharmacologic complications such as excessive phenobarbital dosing as discovered in this case.

## Conclusions

It is often difficult to make treatment decisions based on clinical findings alone in complex patients. MRI with angiogram and MR perfusion imaging are robust imaging modalities that contribute to clinical decision making in patients with Moyamoya and a challenging neurological clinical picture. MR perfusion quantitatively analyzes the status of brain parenchyma, assesses vascular inflow to the brain as well as the relative degree of ischemia/vascular perfusion of the cerebral hemispheres. In conjunction, these modalities can provide vital information in complex cases and assist in clinical decision making and to evaluate the need for revascularization surgery.

## References

[1] Suzuki J, Kodama N (1983). Moyamoya disease-a review. Stroke.

[2] Khan M, Novakovic RL, Rosengart AJ (2006). Intraventricular hemorrhage disclosing neurofibromatosis 1 and moyamoya phenomena. Arch Neurol.

[3] Uchino K, Johnston SC, Becker KJ (2005). Moyamoya disease in Washington State and California. Neurology.

[4] Duan L, Bao XY, Yang WZ (2012). Moyamoya disease in China: its clinical features and outcomes. Stroke.

[5] Kleinloog R, Regli L, Rinkel GJ (2012). Regional differences in incidence and patient characteristics of moyamoya disease: a systematic review. J Neurol Neurosurg Psychiatry.

[6] Ezura M, Takahashi A, Yoshimoto T (1992). Successful treatment of an arteriovenous malformation by chemical embolization with estrogen followed by conventional radiotherapy. Neurosurgery.

[7] Mori N, Mugikura S, Higano S (2009). The leptomeningeal "ivy sign" on fluid-attenuated inversion recovery MR imaging in Moyamoya disease: a sign of decreased cerebral vascular reserve?. AJNR Am J Neuroradiol.

[8] Fukui M (1997). Guidelines for the diagnosis and treatment of spontaneous occlusion of the circle of Willis (Moyamoya'disease). Clin Neurol Neurosurg.

[9] Scott RM, Smith ER (2009). Moyamoya disease and moyamoya syndrome. N Engl J Med.

[10] Calamante F, Ganesan V, Kirkham F (2001). MR perfusion imaging in moyamoya syndrome. Stroke.

